# Perioperative management of von Willebrand patients at the time of implant placement: Case report

**DOI:** 10.1002/ccr3.4755

**Published:** 2021-08-30

**Authors:** Hiroyuki Takashima, Aiji Sato (Boku), Hironori Miyamoro, Shinichiro Kato, Shota Furuno, Yasuyuki Shibuya

**Affiliations:** ^1^ Department of Oral and Maxillofacial Surgery Nagoya City University Graduate School of Medical Sciences Nagoya Japan; ^2^ Department of Anesthesiology Aichi Gakuin University School of Dentistry Nagoya Japan

**Keywords:** a hemostatic factor, factor VIII concentrate (Confact F^®^), von Willebrand disease

## Abstract

In the management of patients with type 1 von Willebrand disease, supplementation with Con Facto F^®^ as well as hemodynamic stabilization with appropriate analgesia and sedation may be important to reduce the risk of bleeding.

## Graphical abstract

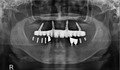

## 1 INTRODUCTION

Von Willebrand disease is a hereditary disease associated with a tendency to bleed, and it is important to reduce the possibility of bleeding in a procedure. This case describes the use of a hemostatic factor, Factor VIII concentrate (Confact F^®^), during a dental procedure involving teeth extraction and implant placement.

Eric von Willebrand first reported Von Willebrand disease (VWD) in 1926.^1^ It is a congenital condition that results in an excessive tendency to bleed owing to the quantitative and qualitative abnormalities of von Willebrand factor (VWF).^2^ In the perioperative management of VWD patients, it is necessary to reduce bleeding by preoperatively replenishing coagulation factors and stabilizing hemodynamics during anesthesia. Herein, we report our experience with a patient with VWD. We performed safe perioperative hemostatic management under intravenous sedation by supplementing with heat‐treated factor VIII concentrate (Confact F^®^) before extracting the teeth and placing implants. Written consent was obtained from the patient for this report.

## 2 CASE REPORT

**FIGURE 1:**
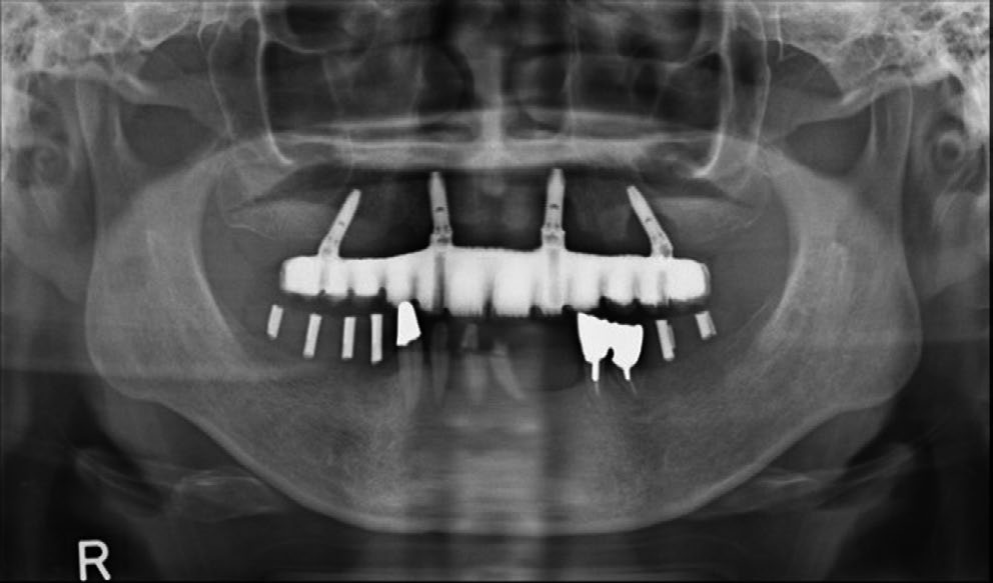
The remaining teeth in his lower jaw were 1, 2, and 3 on the right side and 1, 4, and 5 on the left side. The patient had chronic apical periodontitis and severe periodontal disease

The patient is a 57‐year‐old man (height, 173.5 cm; weight, 61.7 kg), and the remaining teeth in his lower jaw were 1, 2, and 3 on the right side and 1, 4, and 5 on the left side. The patient had chronic apical periodontitis and severe periodontal disease. There was teeth movement, and it was difficult to preserve them. We planned to extract these teeth and place six implants (Figure 1).

A physician had suspected the presence of VWD when the patient was aged 21 years, but no detailed testing or treatment was performed. The family history was not relevant, and the patient was on no current medications.

**FIGURE 2:**
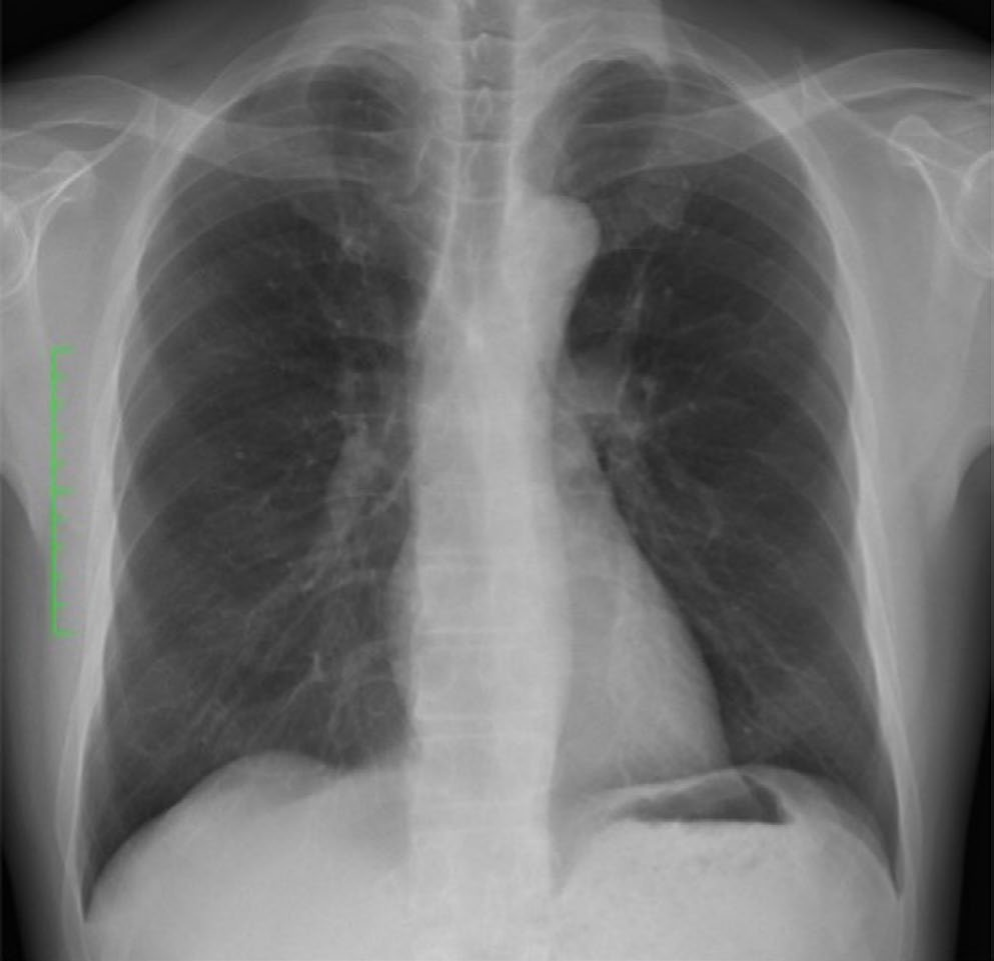
The chest X‐ray was normal with a normal cardiothoracic ratio of 42%

In terms of preoperative chest findings, the chest X‐ray was normal (Figure 2) with a normal cardiothoracic ratio of 42%, and there were no particular issues. Blood test results are shown in Table 1. The platelet count, a hemostatic factor, was 360,000/µl, the activated partial thromboplastin time was 35.4 s, and the prothrombin time was 11.7 s; all were within normal limits. However, coagulation factor VIII was 49%, VWF activity was 24%, and VWF antigen was 26% indicating that the coagulation factor was lower than normal. Based on detailed testing carried out by our hospital's hematology department, the patient was diagnosed with Type 1 VWD, in which there is a quantitative decrease in the VWF.

**TABLE 1:** Blood test results

Items	Results	Reference values
WBC	6.6 × 10^3^/μl	3.6–9.6 × 10^3^/μl
RBC	4.33 × 10^3^/μl	4.00–5.52 × 10^3^/μl
Hb	14.1 g/dl	13.2–17.2 g/dl
Ht	42.5%	40.4%–51.1%
Plt	366 × 10^3^/μl	148–339 × 10^3^/μl
APTT, s	35.4	23–40
PT, s	11.7	9.0–13.0
Coagulation factor VIII	49%	60%–150%
VWF activity	24%	60%–170%
VWF antigen	26%	50%–155%

There was nothing of particular note in terms of family history. Implants in the upper jaw had already been placed at a different hospital, and there had been some difficulty stopping the bleeding when the teeth were extracted. In this case, after consulting with the hematology department, we decided that surgery would be possible by transfusing heat‐treated factor VIII concentrate (Confact F^®^), which contains VWF. This transfusion was done to prevent abnormal bleeding during surgery. Furthermore, considering the risk of bleeding due to fluctuations in hemodynamics, with the patient's consent, we planned to carry out tooth extraction and implant placement under local anesthesia with intravenous sedation.

### 2.1 Anesthetic course

One hour before entering the operating room, 1500 units of Confact F^®^ were transfused. In terms of transfusion volume, although we planned to carry out implant placement, it was determined that the amount of bleeding would not differ greatly from that of tooth extraction alone, so we used 25 IU/kg, basing our calculations on those published in the New England Journal of Medicine (Table 2).^3^ Immediately before surgery, we took another blood sample. We confirmed that VWF activity was 127% and that coagulation factor VIII had increased to 104%. We then proceeded with the surgery. Considering that fluctuations in hemodynamics during surgery promote bleeding, intravenous sedation was performed to stabilize circulation. Oxygen (2 L/min) was administered transnasally, and cefmetazole sodium (1 g) was administered preoperatively to prevent infection. During the operation, we used midazolam iv and propofol (1% Diprivan Injection kit^®^) target‐controlled infusion for continuous intravenous sedation. Also, 2% Xylocaine Dental^®^ with epinephrine 1:80,000 was used for local anesthesia. The operation time was 3 h and 37 min, the anesthesia time was 4 h and 16 min, and blood loss was 405 ml. Although it was a little difficult to stop bleeding during the operation, we completed the procedure with no major problems. No postoperative complications, such as bleeding, were observed, and the prognosis was favorable. One week after the operation, evaluation of the coagulation factor VIII and VWF activity revealed that they had decreased to 48% and 23%, respectively, which were close to their preoperative values (Table 3).

**TABLE 2:** Indicators of VWF replacement

VWF replacement	Replacement volume (IU/kg)		Treatment period (Days)
Blood loss	Minor–moderate	20–40	1–3
	Major	50	7–10
Intervention required (Type)	Tooth extraction	25	1
	Minor operation	30–60	1–5
	Major operation	50–60	7–10
	Delivery	40–50	3–4

**TABLE 3:** Coagulation factors before and after surgery

Item	Before replacement (%)	After replacement (%)	1 week after replacement (%)	Reference values (%)
Coagulation factor VIII	49	104	48	60–150
VWF activity	24	127	23	60–170

## 3 DISCUSSION

Von Willebrand disease is a congenital bleeding disorder inherited in an autosomal dominant manner. This condition consists of a quantitative or qualitative abnormality in the VWF, a hemostatic factor that causes a temporary hemostatic disorder. The VWF functions as an intrinsic coagulation factor that mediates platelet adhesion to subepithelial connective tissue and stabilizes binding to coagulation factor VIII.

A lack or decrease in these functions can cause bleeding. Under the disease classification proposed by the International Society on Thrombosis and Haemostasis, there are the following types of VWD: 1, 2A, 2B, 2M, 2N, and 3.^4^ Type 1 is the most common and features a quantitative deficiency of the VWF but no functional problems. It was previously reported that desmopressin (DDAVP) is effective for hemostasis management in mild‐to‐moderate hemophilia and VWD.^5^ Therefore, DDAVP was used in many cases considering the side effects of replacement therapy of plasma‐derived FVIII concentrates such as the risk of infection from hepatitis virus and AIDS.

However, the increase in deficiency factor activity is uncertain. Although it is used for short‐term hemostasis, it has limited application since it cannot be repeatedly or continuously used. The improved viral inactivation accuracy of Confact F^®^ improves the purity and safety of the drug, and the risk of infection is said to be lower than before.^6^ Although in this case, we planned to perform tooth extraction and implant placement, it was determined that the amount of bleeding would not differ greatly from carrying out tooth extraction alone, so for the transfusion volume, we used 25 IU/kg, basing our calculations on those published in the New England Journal of Medicine (Table 2). The Confact F^®^ Confact F we have used is a high purity plasma‐derived FVIII concentrate. Despite the use of Confact F^®^ replacement therapy, we had some difficulty with hemostasis during the operation. We measured VWF activity before and after Confact F^®^ replacement, but not bleeding time. We should have measured the bleeding time and used antifibrinolytic therapy with trachynesamate, but we did not. This could have reduced the amount of intraoperative blood loss. Furthermore, stabilizing hemodynamics using appropriate analgesia and sedation is also considered extremely important for reducing the risk of bleeding.

## 4 CONCLUSION

In this case, we carried out safe perioperative management under intravenous sedation combined with local anesthesia after preoperatively supplementing with heat‐treated factor VIII concentrate (Confact F^®^) when extracting teeth and placing implants for a Type 1 VWD patient. Stabilizing hemodynamics using appropriate analgesia and sedation is also considered important for reducing the risk of bleeding.

## CONSENT FOR PUBLICATION

The authors obtained written consent for publication from the patient.

## CONFLICT OF INTEREST

None declared.

## AUTHOR CONTRIBUTIONS

All authors contributed to the case. Preoperative management and anesthetic planning were performed by HT, SF, and AS. Anesthetic management was performed by AS, SK, and HM. The first draft of the manuscript was written by AS, and all authors commented on previous versions of the manuscript. YS helped with the supervision of the manuscript and development of the overall periopertative plan. All authors read and approved the final manuscript.

## ETHICAL APPROVAL

Not applicable.

## Data Availability

Not applicable due to patient privacy concerns.
